# Adding insulin glargine vs. NPH insulin to metformin results in a more efficient postprandial *β*-cell protection in individuals with type 2 diabetes

**DOI:** 10.1111/j.1463-1326.2010.01209.x

**Published:** 2010-05

**Authors:** T Forst, M Larbig, C Hohberg, S Forst, S Diessel, M Borchert, W Roth, A Pfützner

**Affiliations:** 1 Institute for Clinical Research and Development, Clinical DepartmentMainz, Germany; 2 Johannes Gutenberg University Mainz, Department of EndocrinologyMainz, Germany; 3 Sanofi-aventis, Medical DepartmentBerlin, Germany

**Keywords:** beta cell stress, insulin glargine, intact proinsulin, NPH insulin

## Abstract

**Aim:**

Postprandial release of intact proinsulin (IP) is an independent marker for *β*-cell dysfunction in patients with type 2 diabetes. This open-label, parallel-group, two-arm, pilot study compared the *β*-cell protective effect of adding insulin glargine (GLA) vs. NPH insulin to ongoing metformin.

**Material and methods:**

Overall, 28 insulin-naive type 2 diabetes subjects (mean ± SD age, 61.5 ± 6.7 years; diabetes duration, 9.8 ± 6.5 years; HbA1c, 7.1 ± 0.5%; BMI, 30.7 ± 4.3 kg/m^2^) treated with metformin and sulfonylurea were randomized to add once-daily GLA or NPH at bedtime. At baseline and after 3 months, subjects received a standardized breakfast, lunch and dinner, with pre- and postprandial blood sampling to measure plasma IP, total insulin and blood glucose (BG).

**Results:**

Insulin dose after 3 months was comparable in both groups (GLA vs. NPH: 23.6 ± 13.4 vs. 23.3 ± 12.7; p = NS ). Both treatments significantly reduced fasting BG levels (GLA: 158 ± 19 to 121 ± 23 mg/dl; NPH: 156 ± 34 to 119 ± 29 mg/dl; both p < 0.01 vs. baseline). Fasting and postprandial BG levels did not differ between groups. IP levels decreased in both groups (p < 0.05 at all timepoints). Although IP release after breakfast did not differ between treatments, GLA induced a greater reduction in IP release after lunch (p = 0.08) and dinner (p = 0.04). Total plasma insulin levels did not differ between groups.

**Conclusions:**

Adding basal insulin to metformin reduces postprandial *β*-cell load. While GLA and NPH had comparable effects at breakfast, GLA reduces *β*-cell stress more effectively at dinner, and with a trend at lunch, most probably because of its longer lasting pharmacodynamic profile.

## Introduction

Increasing *β*-cell stress with subsequent failure to release sufficient amounts of biologically active insulin is thought to precede the deterioration of blood glucose control in individuals with type 2 diabetes treated with oral agents [1,2]. Type 2 diabetes is classically monitored by measurement of laboratory markers, such as HbA1c, glucose, lipids, body mass index and blood pressure. In addition to these traditional laboratory markers, the measurement of intact proinsulin (IP) levels may provide pursuing information with regard to *β*-cell function and disease stage [3,4].

In addition to the much weaker glucose-lowering activity, the increasing IP levels have been reported to be associated with a substantial increase in cardiovascular risk and are now considered to be an independent cardiovascular risk marker in subjects, with and without disturbed glucose metabolism [5–7]. In individuals with type 2 diabetes, the increasing insulin requirements, owing to insulin resistance or excess *β*-cell stimulation by sulfonylurea, result in *β*-cell overload with impaired processing of IP into insulin and C-peptide [8,9]. On the contrary, recent studies have indicated that the early introduction of insulin treatment, even at low doses that are insufficient to restore blood glucose control can reduce *β*-cell stress, improve endogenous insulin processing and might contribute to an improvement in the overall cardiovascular risk profile by reducing circulating IP levels [10–12].

In individuals with type 2 diabetes, a combination of oral antidiabetic drugs (OAD) and basal insulin is commonly initiated after OAD treatment has failed to achieve sufficient metabolic control. In these subjects, basal insulin is usually given in the evening to suppress hepatic gluconeogenesis overnight, while OADs are continued to provide sufficient blood glucose control during the day. Recent studies comparing the long-acting insulin analogue glargine (GLA) vs. NPH insulin in combination with metformin revealed the advantages of insulin GLA, owing to its flat and long-lasting pharmacodynamic profile in patients with type 2 diabetes [13].

The goal of the recent study was to investigate the effect of basal insulin treatment, by adding insulin GLA or NPH insulin to metformin, on postprandial *β*-cell function in patients with type 2 diabetes using a combination of metformin and sulfonylurea.

## Research Design and Methods

Thirty insulin-naive subjects with type 2 diabetes treated with a combination of metformin and sulfonylurea were randomized to receive treatment with insulin GLA or NPH insulin at bedtime along with 1000 mg metformin twice daily. The doses of metformin and sulfonylurea were to be stable over the last 3 months before inclusion in the study. Further inclusion criteria for study participation were an HbA1c level between 6.5 and 8.5%, and an intact fasting proinsulin level between 7 and 20 pmol/l. Subjects were excluded if they had been treated with insulin, peroxisome proliferator-activated receptor-*γ* agonists, glinides or glucosidase inhibitors within the last 4 weeks prior to the screening visit. All other concomitant treatments were kept stable during the study. After the initiation of basal insulin therapy, both insulin treatments were titrated to reach a target fasting glucose level of 100 mg/dl (5.6 mmol/l).

At baseline (before insulin treatment) and 3 months after the initiation of insulin treatment, the subjects visited the study site in the morning after an 8-h overnight fast for a test meal day consisting of three standardized meals. For this test meal day, the subjects were hospitalized in a comfortable environment and an intravenous cannula for blood sampling was placed into a large antecubital or forearm vein. The subjects received a standardized breakfast at 08:00 hours (434 kcal, 26.7 g protein, 15 g fat, 48 g carbohydrates), a standardized lunch at 12 : 00 hours (642 kcal, 48 g protein, 25 g fat, 53 g carbohydrates) and a standardized dinner at 18 : 00 hours (427 kcal, 18 g protein, 23 g fat, 55 g carbohydrates). Blood samples were collected before the test meals and 60 and 120 min after food intake to measure plasma glucose, insulin and IP levels.

The study was conducted in accordance with the Declaration of Helsinki and was approved by the local ethical committee. All subjects gave a written, informed consent.

### Laboratory Analysis

All laboratory measurements were analysed at the Institute for Clinical Research and Development (ikfe GmbH, Mainz, Germany). Blood samples were centrifuged and maintained at −20 °C until analysis. Plasma glucose concentrations were determined by the glucose dehydrogenase method (Super GL, RLT, Möhnesee-Delecke, Germany). Insulin was measured by a chemiluminescence assay (Invitron, Monmouth, UK), which shows a high cross-reactivity with insulin GLA and NPH Insulin. Therefore, the plasma insulin levels given in the study represent the total insulin plasma level comprising endogenous and exogenous insulin. Intact proinsulin was measured using an enzyme-linked immunosorbent assay (LincoResearch, St Charles, MO, USA) and HbA1c was determined by high-performance liquid chromatography (Menarini Diagnostics, Neuss, Germany).

### Safety

Adverse events experienced by subjects during the study were documented by the investigator at each visit.

### Subjects Sample Size Considerations and Statistical Analysis

No clinical information was available for the primary endpoint: the effects of basal insulin supplementation on postprandial IP secretion. Therefore, this study was designed as a pilot study, without confirmatory sample size consideration. The number of participating subjects was estimated based on a previous study investigating the effect of low-dose prandial insulin supplementation on postprandial IP levels [12]. Enrolment of 30 subjects was considered appropriate to obtain data of at least 10 evaluable subjects per treatment arm for the full analysis set.

Results are presented using descriptive summary statistics. All measurements are presented as means ± standard deviation (SD). For the postprandial time course of IP levels, the area under the curve (AUC) was calculated according to the trapezoidal rule. Statistical comparison between baseline and at 3 months of insulin treatment, and between the two treatment groups were performed using the Student's *t*-test (paired and unpaired as appropriate). A two-tailed p < 0.05 was considered statistically significant.

## Results

Thirty subjects were randomized (15 individuals in each study arm). One subject in the NPH insulin group terminated the study by withdrawing informed consent and did not receive any follow-up investigation. One subject in the insulin GLA group was excluded owing to abnormal IP levels. In total, 28 subjects (14 subjects in each group) were included in the per-protocol analysis. Baseline demographic data and clinical characteristics of the two groups are presented in Table 1.

**Table 1:** Clinical characteristics of the study subject.

	Insulin GLA + metformin	NPH insulin + metformin
*N*	14	14
Males/females	12/2	10/4
Age (years)	66.9 ± 6.2	58.0 ± 8.5
Body mass index (kg/m^2^)	30.0 ± 3.7	31.5 ± 4.9
Duration of diabetes (years)	11.6 ± 7.5	8.6 ± 4.7
HbA1c (%)	7.1 ± 0.6	7.1 ± 0.4

GLA, glargine.

Slight but non-significant differences between both study groups were found for age and duration of diabetes. No relevant differences in concomitant medication were found between the two groups.

As shown in Table 1, HbA1c at the start of the study was comparable between the two groups. A slight, but non-significant reduction in HbA1c was observed with both treatments. At the end of the study, the HbA1c levels were comparable between both treatment groups (insulin GLA: 6.9 ± 0.6%; NPH insulin: 6.8 ± 0.7%). Insulin doses at 3 months were comparable with both treatment regimens (insulin GLA: 23.6 ± 13.4 IU; NPH insulin: 23.3 ± 12.7 IU; p = NS ). The postprandial plasma glucose, insulin and IP levels recorded during the test meal days at baseline and after 3 months of insulin treatment are presented in Table 2.

**Table 2:** Blood glucose, insulin and intact proinsulin levels at baseline and 3 months after treatment with insulin GLA or NPH insulin.

			Breakfast			Lunch			Dinner		
			0′	60′	120′	0′	60′	120′	0′	60′	120′
Glucose (mg/dl)	NPH	Baseline	156 ± 34	217 ± 44	204 ± 41	141 ± 34	158 ± 41	140 ± 32	100 ± 16	145 ± 33	147 ± 37
		3 months	119 ± 29*	197 ± 28	183 ± 38	136 ± 38	163 ± 36	146 ± 28	108 ± 15	156 ± 21	154 ± 21
	GLA	Baseline	158 ± 19	233 ± 27	211 ± 29	131 ± 28	142 ± 26	131 ± 20	102 ± 16	147 ± 18	148 ± 19
		3 months	121 ± 23*	200 ± 29	181 ± 28*	117 ± 24	160 ± 23	151 ± 23	108 ± 19	159 ± 21	158 ± 26
Insulin (mU/l)	NPH	Baseline	18.6 ± 11.3	56.2 ± 22.2	64.0 ± 25.5	39.6 ± 20.7	52.3 ± 22.5	46.6 ± 17.6	24.0 ± 16.9	35.8 ± 19.1	39.5 ± 22.3
		3 months	20.0 ± 9.6	46.8 ± 20.7*	51.6 ± 27.5*	33.0 ± 16.7	42.5 ± 15.6*	33.2 ± 13.7*	17.6 ± 9.3*	28.9 ± 11.9*	29.0 ± 10.8*
	GLA	Baseline	14.7 ± 8.3	55.5 ± 27.7	66.7 ± 43.1	42.4 ± 23.2	44.3 ± 21.2	38.8 ± 30.4	17.5 ± 8.8	28.0 ± 13.1	31.2 ± 15.2
		3 months	19.4 ± 7.8*	44.9 ± 16.7	49.8 ± 24.8	24.6 ± 11.1*	36.9 ± 15.7	31.5 ± 13.7	16.2 ± 6.2	25.7 ± 9.3	26.7 ± 8.9
Proinsulin (pmol/l)	NPH	Baseline	20.5 ± 12.7	33.3 ± 20.5	48.3 ± 27.3	47.6 ± 27.4	54.4 ± 31.4	57.5 ± 33.4	38.5 ± 24.7	41.1 ± 22.8	45.2 ± 26.9
		3 months	7.2 ± 6.3*	13.5 ± 9.5*	23.1 ± 13.0*	22.3 ± 13.7*	25.5 ± 14.7*	24.4 ± 14.2*	15.0 ± 11.4*	17.8 ± 12.0*	20.2 ± 12.5*
	GLA	Baseline	16.5 ± 6.1	27.1 ± 7.5	44.9 ± 15.6	48.9 ± 12.9	48.6 ± 14.7	48.0 ± 16.6	29.7 ± 10.4	31.4 ± 11.0	36.4 ± 11.9
		3 months	5.7 ± 2.8*	12.8 ± 5.8*	23.1 ± 13.0*	17.7 ± 7.9*	20.1 ± 9.1*	20.7 ± 6.7	10.6 ± 5.3*	12.5 ± 5.7*	14.5 ± 5.9*

Mean ± standard deviation. GLA, glargine.

* p < 0.05 at 3 months vs. baseline.

A significant reduction in fasting blood glucose levels from baseline to endpoint was observed for both insulin treatments, but no consistent change in the time course of postprandial glucose levels was observed during the study period. Fasting blood insulin concentrations increased significantly after adding insulin GLA, but not with NPH insulin. No other consistent difference in the time course of total (endogenous and exogenous) blood insulin concentrations from baseline to endpoint or between the two basal insulin treatments were observed.

**Figure 1:**
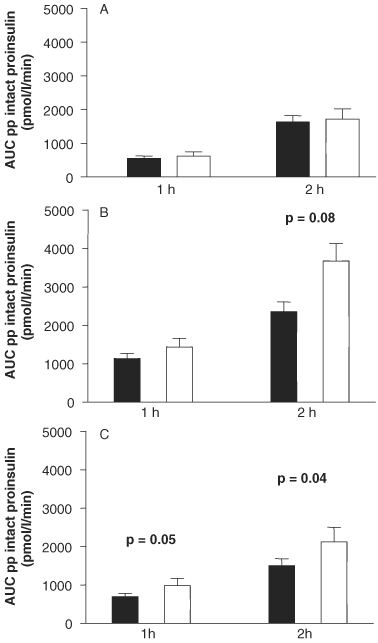
Area under the curve (AUC) for 1- and 2-hour postprandial intact proinsulin levels after 3 months of treatment with adding evening insulin glargine vs. NPH insulin to metformin 1000 mg twice daily at breakfast (A), lunch (B), dinner (C) (white: NPH Insulin, black: Insulin Glargine).

As shown in Table 2, adding once-daily basal insulin to metformin resulted in a remarkable decrease in fasting and postprandial IP levels at all test meals. Figure 1 shows the AUC for postprandial IP levels at 1 and 2 h after breakfast, lunch and dinner.

While the total release of IP after breakfast was comparable between the two insulin treatments, the postprandial release of IP tend to be lower after lunch, and was significantly lower after dinner with insulin GLA when compared with NPH.

Overall, 12 individuals (six people in each treatment group) reported a total of 16 adverse events (6 and 10 events in the insulin GLA and NPH insulin group, respectively). The most frequent type of adverse events in the NPH insulin group was musculoskeletal and connective tissue disorders (three patients reporting four events) and in the insulin GLA group was infections (two patients reporting one event each). Hypoglycaemia was reported by two patients (one event each), both occurring in the NPH insulin arm. On the contrary, the only adverse event that was considered serious (a case of Meniere's disease) occurred in the insulin GLA group.

## Conclusions

In individuals with type 2 diabetes, OAD treatment over time is often followed by a deterioration in *β*-cell function, as indicated by a sustained loss of blood glucose control [1,2]. Early *β*-cell dysfunction may have important physiological implications and may serve as a target for novel treatment strategies for diabetes mellitus [14]. Even before *β*-cell failure becomes apparent with a clinically detectable deterioration in metabolic control, there is a marked increase in release of IP, which was reported to be an early marker for *β*-cell dysfunction [3,15,16]. Furthermore, stimulation of the *β* cell by sulfonylureas might impair the conversion rate from IP to insulin and C-peptide [8,9]. On the contrary, the initiation of insulin in individuals with type 2 diabetes, even if it is not titrated to reduce blood glucose levels, was found to reduce *β*-cell stress and improve the conversion rate of IP [11,12]. Once OADs are unable to maintain glycaemic control, insulin treatment in type 2 diabetes is often initiated by basal insulin supplementation in the evening, while maintaining OADs during the day. The main finding of our study is that in subjects with inadequate control with sulfonylurea and metformin treatment, the initiation of basal insulin treatment not only offers a reduction in fasting glucose levels but also improves *β*-cell function during the day. Although a reduction in postprandial *β*-cell stress was observed in our study, there was no significant improvement in postprandial blood glucose levels after breakfast, lunch and dinner. Therefore, this finding suggests that the initiation of insulin supplementation in individuals with type 2 diabetes might evolve *β*-cell protective effects which go beyond the regulation of blood glucose.

In addition to its use as a marker for *β*-cell damage, elevated IP levels were shown to stimulate adipocytes and decrease circulating adiponectin levels. Intact proinsulin has been shown to increase plasminogen activator inhibitor-1 levels, which is associated to an increase in carotid intima media thickness [17]. In addition, elevated IP levels have been shown to predict coronary atherosclerosis [18] and the risk of cardiovascular events in subjects with and without diabetes [5,6,19,20]. Furthermore, we recently reported a postprandial increase in IP levels in non-diabetic subjects with increased cardiovascular risk [7]. In a clinical study, proinsulin administration for at least one year was associated with an increased rate of cardiovascular events [21]. Therefore, our findings of a marked overall decrease in fasting and postprandial IP levels after the initiation of basal insulin treatment might have important implications not only for metabolic control, but also for cardiovascular risk reduction in individuals with type 2 diabetes.

Insulin GLA is a long-acting human insulin analogue with a longer time–action profile and no pronounced peak of action when compared with NPH insulin [22]. In a recent study, treatment with insulin GLA in combination with OADs achieved better postprandial metabolic control when compared with NPH insulin in combination with OADs [13]. In our study population, no significant differences in glucose control were observed between the two insulin formulations. On the contrary, treatment with insulin GLA in combination with metformin revealed a more pronounced reduction in postprandial release of IP after lunch and dinner when compared with NPH insulin. Owing to the shorter time–action profile of NPH insulin, the rate of insulin release from the subcutaneous tissue depot is more rapid, which exhausts the supply more quickly and, thus, requires earlier endogenous insulin release. The greater demand on *β* cell will become evident, particularly after a meal, when the requirements for insulin are high. In individuals with type 2 diabetes who have barely compensated *β*-cell function, this will lead to a greater release of IP from the exhausted *β* cells [23–25]. Despite comparable glucose control, the prolonged pharmacodynamic profile of insulin GLA results in stronger *β*-cell protection, lasting over 24 h.

The comparable total plasma insulin levels found in both treatment groups indicate that, in NPH insulin-treated subjects, enhanced endogenous insulin release was able to compensate for the shorter time–action profile of NPH insulin, thereby keeping blood glucose levels comparable between the study groups. Nevertheless, the greater demand on the *β* cells in NPH insulin-treated subjects will be followed by an increase in the release of IP, particularly after meals, as observed in our study.

A potential limitation of our findings is that this was an exploratory pilot study to evaluate the protective effects on the *β* cell by initiating basal insulin therapy with metformin in individuals with type 2 diabetes, pretreated with OADs (sulfonylurea in combination with metformin). Further studies are needed to investigate if the effect of basal insulin supplementation will translate into longer *β*-cell survival or a reduction in cardiovascular risk. In addition, our study only compared once-daily application of insulin GLA, with once-daily injection of NPH insulin. Most probably, NPH insulin administered twice daily will result in postprandial IP levels that more closely match those achieved with once-daily insulin GLA.

In conclusion, the initiation of basal insulin in combination with metformin results in an overall improvement of *β*-cell function, as indicated by a reduction in the fasting and postprandial release of IP from the *β* cells. Because of the protracted pharmacokinetic profile of insulin GLA compared with NPH insulin, treatment with insulin GLA may offer more prolonged *β*-cell preservation when compared with NPH insulin applied once daily.
